# Prosthetic journey of magnets: a review

**DOI:** 10.25122/jml-2020-0012

**Published:** 2023-04

**Authors:** Satya Sai Sruthi Yalamolu, Lakshmana Rao Bathala, Satyanarayana Tammineedi, Sri Harika Lakshmi Parvathi Pragallapati, Chakradhar Vadlamudi

**Affiliations:** 1 Department of Prosthodontics, Lenora Institute of Dental Sciences, Rajahmundry, Andhra Pradesh, India

**Keywords:** magnets, retention, overdenture, maxillofacial prosthesis, prosthodontics, denture, implant, GPT – glossary of prosthodontic terms, Sm-Co – samarium cobalt, PRISMA – Preferred Reporting Items for Systematic Review and Meta-Analyses

## Abstract

Magnets have been widely used in dentistry as a means of retention in various prosthodontic applications. This review summarizes the historical background, types, and modes of action of magnets in dentistry, including their uses in conventional removable prostheses, sectional dentures, overdentures, maxillofacial prostheses, and implant-supported prostheses. A comprehensive electronic literature search was performed through multiple databases, including Medline via Pubmed, Wiley Online Library, Ebscohost, Science Direct, and Google Scholar. We used the following keywords: "magnets", "retention", "overdenture", and "maxillofacial prosthesis", with a focus on articles published between October 1953 and March 2016. We found 20 articles, and 16 were selected for inclusion in this review based on their relevance to the topic at hand. Recent advancements in magnetic technology have resulted in newer magnets that exhibit superior biological compatibility and corrosion resistance. These properties have made magnets an effective retentive aid intra- and extra-orally.

## INTRODUCTION

The success of a prosthetic treatment depends not only on the functional integration of the prosthesis with the patient’s oral functions but also on their psychological acceptance of the prosthesis. These parameters require that patients perceive their prosthesis as stable or securely retained during function while also meeting their esthetic and psychodynamic requirements regarding the prosthesis's impact on their facial appearance and sense of well-being. Retention, which is the "quality inherent in the dental prosthesis acting to resist the forces of dislodgment along the path of placement", is a crucial factor in achieving these goals [1]. Factors affecting the retention are (1) anatomical (i.e., size and quality of denture bearing area, tongue size, palatal vault), (2) physiological (i.e., saliva quantity and its quality, orofacial musculature), (3) physical (i.e., adhesive and cohesive property, interfacial surface tension, capillarity, pressure of the atmosphere, gravity), and (4) mechanical (i.e., tissue or bony undercuts, denture extensions, denture adhesives, retentive aids like a suction cup, magnets, implants) [1]. Many attachments and retentive devices used to improve the mechanical retention of the prosthesis require sophisticated equipment and adjuncts, with innovative chairside and laboratory techniques. Magnets have become increasingly popular and are now extensively used in various fields of dentistry, including prosthodontics. Initially, their usage was limited due to the inaccessibility of mini compact magnets. However, the launch of bijou (small) magnets with stronger attractive forces have increased their popularity and use in prosthetic dentistry [2,3].

While the long-lasting durability of magnets presented challenges in the early days of usage, recent studies have shown that advances in the composition of different magnetic attachments have sustained their attractive forces, regardless of subjective variables. The main objective of this review was to provide a comprehensive overview of the history of magnets, the types of magnets available, their applications in prosthodontics, and recent advancements in the science of materials [2]. By examining the current state-of-the-art and future directions of magnet-based prosthetics, we hope to contribute to the ongoing efforts to improve patient outcomes in prosthodontics.

## MATERIAL AND METHODS

### Search protocol

A computerized literature search was conducted to identify relevant studies on the use of magnets in prosthodontics. The search was performed using the keywords "magnets", "retention", "overdenture", "maxillofacial prosthesis", "prosthodontics", "denture", and "implant" in the following databases: Medline via PubMed, Science Direct, Scopus, Wiley Online Library, Ebscohost, Web of Science, and Google Scholar. The search period was limited to articles published between October 1953 and March 2016.

### Eligibility criteria

We included full-text articles written in English that met the following criteria: relevance to the keywords mentioned above and availability of the full text. Articles written in languages other than English, those with only abstracts available, and smaller studies were excluded from the analysis.

### Data extraction

**Figure 1:**
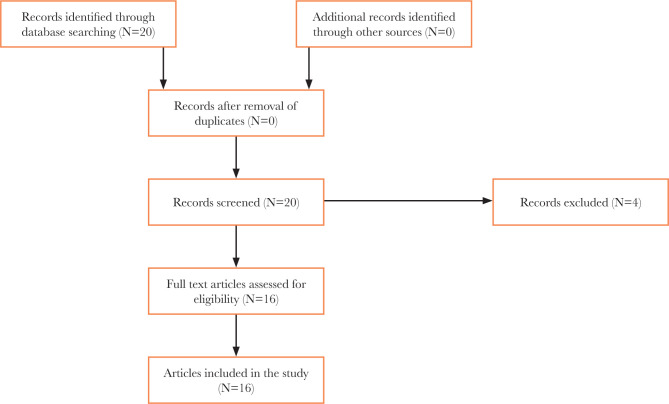
PRISMA flowchart for selection of the articles.

We reviewed the full text of the articles to extract relevant data. The data extraction process focused on the types of magnets available, their applications in prosthodontics, and recent advancements in the science of materials. A total of 20 articles were initially identified through the literature search, and after excluding 4 articles that did not meet the inclusion criteria, 16 articles were finally selected (see Figure 1 for a summary of the search and selection process). The data extracted from these 16 articles were carefully analyzed to provide a comprehensive overview of the topic.

## Results

### History of magnets in prosthodontics

Historically, magnets were rarely used in medical literature in the 19th century, but they became more extensively adopted in orthopedic surgeries to treat non-unionized fractures [4]. The introduction of magnets into the field of dental science began in the 1940s when Freedman attempted to multiply the retentive nature of the denture in patients with severely resorbed edentulous mandibular arches. Table 1 provides a summary of the historical milestones in the development of magnets in dentistry [5]. The classification of magnets is showcased in Table 2.

**Table 1:** Historical advances in the use of magnets in prosthodontics.

Author & year	Work
**Freedman (1940)**	Used improved retention of the mandibular denture in severely resorbed ridges.
**Freedman (1953)**	Used magnets in repulsion to maintain and improvise the seating of complete dentures.
**Nadeau (1956)**	Used extra-oral and intra-oral prostheses connected by magnets.
**Behrman SJ (1960)**	Presented techniques for implanting magnets in the jaws to enhance the retention of prostheses.
**Robinson (1963)**	Used to retain surgical prostheses for patients who have a radical surgical treatment. Described a method of constructing a two-sectioned intra-oral prosthesis using magnets.
**Becker and Hoffer (1967)**	Developed a new magnetic alloy of Co_5_Sm.
**Robert J Connor (1977)**	Stated the possibility of effectively sealing an Sm-Co magnet from the in vivo environment by using a protective coating composed of polytetrafluoroethylene and pyrolytic graphite.
**Sasakietal H (1984)**	Stated the use of Sm-Co magnets to retain sectional prostheses.
**Lemon JC, Brignoni RA (2004)**	Showed that microwave irradiation reduced the magnetism of Sm-Co by 12%.

**Table 2:** Multi-factor classification of magnets.

**Alloy used**	Magnets restraining cobalt (e.g., Alnico V, Co_5_Sm, Alnico, Co-Pt)
	Magnets non-restraining cobalt (e.g., Samarium iron nitride, Nd-Fe-B)
**Ability to retain their magnetic properties (flexibility/solidity)**	Soft (easy to magnetize and does not last long). For example, Pd-Co-Pt alloy, Pd-Co alloy, Pd-Co-Cr alloy, Pd-Co-Ni alloy, magnetic stainless steels, Cr-Molybdenum alloy, Permendur (an alloy of Fe-Co)
	Hard (magnetism lasts long) (e.g., Co_5_Sm, Co-Pt, Nd-Fe-B, Alnico alloys)
**Coating (Titanium, stainless steel, Palladium)**	Surface Coated
	Surface Uncoated
**Class of magnetic field**	Open magnetic field
	Closed magnetic field: a) rectangular sandwich design; b) circular sandwich design
**Number of magnets in the system**	Singular
	Paired sets
**Polar arrangement**	Reversed polar arrangement
	Non-reversed polar arrangement

### Components and specifications of magnets

The standard magnetic retention unit is a two-component system, including the retentive component and the keeper component. The retentive component consists of a set of two magnets, a keeper attached to them, and two end plates that provide a protective cover for the magnet faces. The retentive component is oval in shape, 5mm long, 3.2mm wide, and 3mm high. The keeper component is an oval, detachable, magnetized disk prepared with a magnetizable alloy, which acts as a magnet (induced magnet) when it comes in contact with the magnetic retention of the element. The alloy used is Pd-Co-Ni or stainless-steel alloy.

Recently, a new permanent magnetic alloy of neodymium-iron-boron, which has 20% more magnetic strength than cobalt samarium per unit volume, has been introduced. The neodymium-iron-boron disk is preformed and cemented into the keeper, measuring 5mm in length, 3.2mm in width, and 1.2mm in thickness. A preformed disk with one face measuring 5mm in length, 3.2mm in width, and 1.2mm in thickness and another measuring 6mm in length, 4mm in width, and 1.2mm in thickness is then screwed onto the keeper [4].

### Physical and biological properties of magnets

The biological properties of magnets have been shown to produce cytotoxic corrosive products. However, the encapsulation of magnets can overcome this issue [4]. In terms of mechanical properties, magnets have a density of 8.1G/cm3, high solidity, and almost no elongative properties, resulting in slight brittleness [4]. The thermal properties include the thermal expansion coefficient of magnets, comparable to that of Co-Cr alloy (i.e., 12.6*10-6/C) [4]. Retentive forces, also known as breakaway loads, are estimated between the retentive and keeper components with diverse keeper thicknesses. While the attached keeper thickness is maintained constant, the detachable keeper thickness ranges from 0.3 to 2mm [6].

### The manufacturing process of rare earth magnets

Rare earth magnets are typically fabricated using a sintering process (i.e., a fine alloy powder pressed together into a mold, which forms a non-porous cohesive matter).

### Patterns

An open magnetic field is made of cylindrical magnets with open ends, either singular or pairs. A closed magnetic field comprises paired magnets and an attached and detachable keeper, which can be oval or circular. The paired magnets may vary in width and height, with the most common being 2.5mm in width and 1.5mm in height or 3mm in width and 2.5mm in height. Key features of ‘keepers’ include magnetizability, low-coerciveness, and end plates made of stainless steel, which join the unlike poles of a magnet. These ‘keepers’ help provide a closed field pathway and eliminate the external field.

The first closed field pattern to be developed was the split pole pattern, which consists of two magnets with adjacent opposite poles. A soft magnetic keeper adheres to the peak of the magnets, and another keeper is assembled into the root. While various patterns exist, circular sandwich-type designs with a closed field have been found to have the highest retentive capacity [6]. Magnetic retention systems offer several advantages such as ease of placement, automatic reseating, strong attractive forces with a small size, ease of replacement, ability to be assembled in the prosthesis, diminished need for parallel abutments, ease of cleaning, cabability to engage soft tissue undercuts, and to use roots with less than 3mm bony support as abutments with magnetic devices. Additionally, magnetic retention systems do not apply direct stress to root abutments. However, they also have some disadvantages, such as low corrosive resistance, cytotoxic effects due to the use of corrosive products, and a high cost, although it is still lower than that of implants [6].

### Development of materials

Over the past century, significant advances have led to the development of magnetic materials that have been quickly incorporated into the field of dentistry. The primary material used is the rare earth material neodymium iron boron (Nd-Fe-B) alloy, the most accessible magnetic material. Another material used is samarium cobalt (Sm-Co), otherwise known as RE alloy. Prior to the development of rare earth magnets, the main materials used were Alnicos-alloys formed on cobalt, aluminum, nickel, and cobalt platinum (Co-Pt) magnets.

A promising development in magnets is samarium iron nitride due to its resistance to demagnetization, peak magnetization, and higher resistance to temperature and corrosion compared to Nd-Fe-B. However, this material is still under research [7].

### The effects of magnets on tissues and precautionary measures

In dentistry, magnets can potentially lead to tissue injury in two main ways. Firstly, the continuous magnetism encircling the tissues can cause physical effects. Studies have been conducted on the magnetic effects, but the results are conflicting. However, implementing magnetic retention systems in dentistry has not shown any deleterious effects on the tissues. It has been observed that the closed magnetic field performs better in terms of tissue compatibility compared to the open system. The second way magnets can lead to tissue injury is through the chemical effects of alloys and their corrosion products. Cobalt and samarium salts are not typically considered toxic. Cerium oxalate is another rare earth salt that contains samarium and has been recommended as a treatment option for seasickness in low dosages (up to 1gm/day).

### Modes of action

#### Repulsive properties of magnets

Alnico-type magnets were first used in prosthetic dentistry due to their repulsive properties of the like poles for stabilizing complete dentures. However, they were discontinued for dental use due to their bulky size needed for strength. Magnets were embedded in the molar regions in the complete denture bases, with like poles facing each other. As the patient tries to close the jaws, the repulsive forces of the like poles of magnets help seat the prosthesis on the ridges. However, the persistent repelling force can promote the resorption of the alveolar bone, leading to a drastic loss of stability during jaw opening [7].

#### Attraction properties of magnets

In the early 1960s, the attractive property of magnets was used for denture retention. The initial attempts consisted of implanting Alnico V magnets in the soft tissue of the edentate lower arch and the tissue surface of the prosthesis, with unlike poles facing each other [7]. In prosthodontics, magnets are used with implants or in a conventional removable prosthesis, sectional dentures, overdentures, or maxillofacial prostheses.

### Conventional removable prosthesis

For conventional removable prostheses, magnets can function in either repulsive or attractive modes. In repulsive action mode, magnets are placed in the molar regions of the dentures, with like poles facing each other. This arrangement creates repulsive forces that allow the dentures to be seated on their alveolar ridges. In the attractive mode, the keeper and retentive components are placed in the soft tissue and the tissue surface of the denture, respectively.

### Sectional dentures

In patients with limited mouth opening, such as those with microstomia, it is difficult to fabricate and place the prosthesis. To address this, sectional dentures or prostheses in two parts are used. However, the main challenge with sectional dentures is the retention of the two sections of the prosthesis as a single unit in the oral cavity. Magnets can help overcome this problem by retaining the two parts of the denture. In the attractive mode of action, magnets are arranged so that each sectional prosthesis has a magnet with unlike poles attracting each other to retain the sectional dentures.

### Overdentures

The magnetic retention unit contains a denture-retention element and a demountable keeper element. The denture-retention element has a set of cylindrical axially magnetized cobalt-samarium magnets with opposite poles adjacent to each other [5]. The root faces of teeth are used for selecting the magnets. In contrast, Co_5_Sm conventional magnets can be used in mini sizes due to their extreme coerciveness. However, this can cause reduced strength of the magnetic field. The magnets used in denture-retention elements have a diameter of 1.5mm, while the overall height of the retention unit is 2.7mm. This height includes the 1.5mm height of the magnets and the 1.2mm thickness of the keeper element. [8].

### Maxillofacial prosthesis

To retain the maxillofacial prosthesis, the attractive mode of action is typically used by placing magnets on the soft tissue and the tissue side of the prosthesis, with unlike poles facing each other. To restore large maxillofacial defects, sectional prostheses are fabricated, where the magnets can be placed to retain the two sections of the prosthesis together.

### Use along with implants

Implants consist of a magnet cap threaded onto the abutment and a magnet placed onto the tissue surface of the prosthesis. This technique is particularly useful when the abutments are not parallel, in orbital and auricular prosthesis with or without a bar clip system, and shallow defects with insufficient space for a bar and clip attachment [9].

### Positioning of magnets

In overdentures, when the repulsive mechanism is intended to be used, magnets are positioned posterior to the second molar on the denture-bearing areas and anterior to the first premolar. When the attractive mechanism is used, the magnets are placed on the tissue side of the canine and the first and second molars [8].

Maxillofacial prostheses often rely on the attractive mechanism and are placed where undercuts are not present and in the most accessible areas, based on the clinical condition. When used with implants, the abutment should have a magnet cap threaded, and the prosthesis tissue surface should have a magnet [9].

## DISCUSSION

Since the early 1980s, magnetic attachments have been used to fabricate overdentures. The evolution of new-era cobalt and rare earth alloys with magnetic properties expanded the potential uses of magnets as retentive support in various types of dental prostheses, including non-fixed complete and partial dentures, overdentures, fixed partial dentures, and two-part dentures. Research has been conducted to investigate the potential adverse effects of magnetic fields on the body's tissues, with closed-field magnet systems found less likely to cause harm than open-field magnets. In the case of overdenture, a detachable keeper is either cemented or screwed to a prepared tooth structure. The retention element consists of a set of magnets and a fixed keeper that attach the keeper to the root and seats the denture through the attractive nature of magnets. This type of arrangement helps to increase existing retention.

In 1953, Behrman SF et al. [10] used magnets to increase the stability and retention of the prosthesis by positioning various poles of magnets in the prosthesis and the arch. Freedman H [11] in 1953, and Winkler S et al. [12], in 1967, proposed positioning the identical poles by using repulsive forces in both upper and lower dentures to achieve retention and stability. Boucher LJ et al. [13], in 1966, showcased the bridging of the prosthesis (intraoral or extraoral), using magnets to reinstitute extraoral defects.

Burns et al. [14] in 1995 and Naert et al. [15] in 1997 elucidated that the retention force of magnetic attachments is maintained for longer, but the force is weaker when compared to other retentive mechanical aids. Magnetic attachments apply an attractive force of more than 400 gf. In 2000, Setz et al. [16] launched the concept that the attractive force remains constant and that the main causative factor for a decreased retention force is the constant gap between the assembly, keeper, and attractive force. In 1999, Riley et al. [17] reported that the failure of magnets as attachments is often caused by an epoxy seal or encapsulating material breakdown and suggested that addressing these issues could prolong the lifespan of magnets. The study also highlighted that these magnets are not commonly used as attachments in the West due to decreased attractive force and the risk of corrosion resulting from sealing failure.

In contrast, Thean et al. [18] reported, in 2001, that using laser welding for sealing can reduce corrosion. Mantri SS et al. [19], in 2013, used magnets to retain the two-piece maxillofacial prosthesis and rehabilitate the mandibular segmental defect. In 2016, Leem H et al. [20] concluded that certain variables (e.g., sex of patients, abutment type, the position of attachments, types of overdentures, attachments class, and opposing dentition classification) do not alter the attractive force applied by the magnets.

However, the long-term durability of magnets remains a significant concern. Further research is needed to evaluate their compatibility with oral tissues and to develop methods for enhancing the longevity of the attachment system. These efforts may ultimately result in the development of magnetic materials that are highly resistant to the effects of the oral environment, providing more reliable and long-lasting solutions for a variety of dental applications.

## CONCLUSION

The simplicity of clinical and laboratory procedures has fueled the growing interest in magnetic retention as a promising option for enhancing the stability of dental prostheses. Although the long-term durability of magnets presented a problem in earlier days, recent studies have shown that their attractive force is not altered by various subjective variables. Current research has provided dentistry with newer magnets with excellent biological compatibility, corrosion resistance, sealing ability, closed-field magnetism, and enhanced magnetic force, even with smaller sizes. Therefore, magnets can be used as an effective retentive aid both intra- and extra-orally.

## Data Availability

Further data is available from the corresponding author on reasonable request.
